# Semi-Quantitative Multiplex Profiling of the Complement System Identifies Associations of Complement Proteins with Genetic Variants and Metabolites in Age-Related Macular Degeneration

**DOI:** 10.3390/jpm11121256

**Published:** 2021-11-25

**Authors:** I. Erkin Acar, Esther Willems, Eveline Kersten, Jenneke Keizer-Garritsen, Else Kragt, Bjorn Bakker, Tessel E. Galesloot, Carel B. Hoyng, Sascha Fauser, Alain J. van Gool, Yara T. E. Lechanteur, Elod Koertvely, Everson Nogoceke, Jolein Gloerich, Marien I. de Jonge, Laura Lorés-Motta, Anneke I. den Hollander

**Affiliations:** 1 Department of Ophthalmology, Donders Institute for Brain, Cognition and Behaviour, Radboud University Medical Center, 6525 GA Nijmegen, The Netherlands; erkin.acar@radboudumc.nl (I.E.A.); Eveline.Kersten@radboudumc.nl (E.K.); Bjorn.Bakker@radboudumc.nl (B.B.); Carel.Hoyng@radboudumc.nl (C.B.H.); yara.lechanteur@radboudumc.nl (Y.T.E.L.); lauraloresmotta@protonmail.com (L.L.-M.); 2 Laboratory of Medical Immunology, Department of Laboratory Medicine, Radboud Institute for Molecular Life Sciences, Radboud University Medical Center, 6525 GA Nijmegen, The Netherlands; Esther.Willems1@radboudumc.nl (E.W.); marien.dejonge@radboudumc.nl (M.I.d.J.); 3 Radboud Center for Infectious Diseases, Radboud University Medical Center, 6525 GA Nijmegen, The Netherlands; 4 Translational Metabolic Laboratory, Department of Laboratory Medicine, Radboud Institute for Molecular Life Sciences, Radboud University Medical Center, 6525 GA Nijmegen, The Netherlands; Jenneke.Keizer-Garritsen@radboudumc.nl (J.K.-G.); Else.Kragt@gmail.com (E.K.); Alain.vanGool@radboudumc.nl (A.J.v.G.); Jolein.Gloerich@radboudumc.nl (J.G.); 5 Department for Health Evidence, Radboud Institute for Health Sciences, Radboud University Medical Center, 6525 GA Nijmegen, The Netherlands; tessel.galesloot@radboudumc.nl; 6 Roche Pharma Research and Early Development, Roche Innovation Center Basel, 124 Grenzacherstrasse, 4070 Basel, Switzerland; sascha.fauser@roche.com (S.F.); elod.koertvely@roche.com (E.K.); everson.nogoceke@roche.com (E.N.); 7 Department of Human Genetics, Donders Institute for Brain, Cognition and Behaviour, Radboud University Medical Center, 6525 GA Nijmegen, The Netherlands

**Keywords:** age-related macular degeneration, AMD, complement system, semi-quantitative multiplex profilin, mass spectrometry, C4, vitronectin, factor I, genetic variants, metabolites, HDL

## Abstract

Age-related macular degeneration (AMD) is a major cause of vision loss among the elderly in the Western world. The complement system has been identified as one of the main AMD disease pathways. We performed a comprehensive expression analysis of 32 complement proteins in plasma samples of 255 AMD patients and 221 control individuals using mass spectrometry-based semi-quantitative multiplex profiling. We detected significant associations of complement protein levels with age, sex and body-mass index (BMI), and potential associations of C-reactive protein, factor H related-2 (FHR-2) and collectin-11 with AMD. In addition, we confirmed previously described associations and identified new associations of AMD variants with complement levels. New associations include increased C4 levels for rs181705462 at the *C2*/*CFB* locus, decreased vitronectin (VTN) levels for rs11080055 at the *TMEM97*/*VTN* locus and decreased factor I levels for rs10033900 at the *CFI* locus. Finally, we detected significant associations between AMD-associated metabolites and complement proteins in plasma. The most significant complement-metabolite associations included increased high density lipoprotein (HDL) subparticle levels with decreased C3, factor H (FH) and VTN levels. The results of our study indicate that demographic factors, genetic variants and circulating metabolites are associated with complement protein components. We suggest that these factors should be considered to design personalized treatment approaches and to increase the success of clinical trials targeting the complement system.

## 1. Introduction

Age-related macular degeneration (AMD) is a major cause of vision loss among the elderly in the Western world. Due to increased ageing of the population, the number of individuals affected by AMD worldwide is expected to rise from 200 million currently to approximately 288 million by 2040 [1]. Early forms of AMD are characterized by the accumulation of drusen between the retinal pigment epithelium (RPE) and Bruch’s membrane. As the disease progresses, the drusen expand and coalesce, leading to two types of advanced stages. Geographic atrophy (GA) is defined by atrophy of photoreceptors, RPE and choriocapillaris in the macula, leading to progressive and irreversible loss of central vision. Neovascular AMD (nAMD) is characterized by the abnormal ingrowth of new blood vessels from the choriocapillaris into the sub-RPE or subretinal space. These aberrant vessels are fragile and tend to leak fluid and blood, causing rapid and severe vision loss. Neovascularization in nAMD can effectively be treated with intravitreal injections of anti-vascular endothelial growth factor (VEGF), but no approved treatment is currently available to prevent degeneration in GA.

AMD is a multifactorial disease caused by a combination of environmental and genetic risk factors. Increasing age, female sex, smoking and a high body-mass index (BMI) are associated with an increased risk for AMD, while use of antioxidants decreases the risk of AMD disease progression [2,3,4,5]. Genome-wide association studies have identified the complement system, extracellular matrix remodelling and lipid metabolism as main AMD disease pathways [6]. The strongest independent genetic associations were reported for the common p.Tyr402His (rs1061170) variant in the complement factor H (*CFH*) gene [7,8,9,10] and for a common variant at the *ARMS2*/*HTRA1* locus [11,12,13]. Since then, eight additional genetic variants at the *CFH* locus have been independently associated with AMD. In addition, four variants in or near the complement factor B (*CFB*) and complement component 2 (*C2*) genes, three variants in or near the complement component 3 (*C3*) gene, two variants in or near the complement factor I (*CFI*) gene, one variant in the complement component 9 (*C9*) gene and one variant near the vitronectin (*VTN*) gene have been associated with AMD [6,14,15]. These variants include intergenic, intronic and coding variants that either confer an increased or decreased risk for AMD. Coding variants in these genes include the variants p.Gly119Arg (rs141853578) in *CFI*, p.Arg102Gly (rs2230199) and p.Lys155Gln (rs147859257) in *C3*, and p.Pro167Ser (rs34882957) in *C9*, which are associated with an increased risk of AMD. Two coding variants in the *CFB* gene, p.Leu9His (rs4151667) and p.Arg32Gln (rs641153), are associated with a decreased risk for AMD. In addition to these variants that are individually associated with AMD, an aggregated effect of rare coding variants in the *CFH*, *CFI*, *C3* and *C9* genes has also been reported to associate with AMD using gene-based approaches [6,16,17,18,19].

An important role of the complement system in AMD already emerged from earlier studies demonstrating that complement components are constituents of drusen [8,14,20,21,22]. In addition, altered concentrations of complement components have been detected in the systemic circulation of AMD patients. Several have studies demonstrated that complement activation markers such as C3a, C3d and C5a are elevated in AMD patients compared to controls [23,24,25]. Moreover, several genetic variants in or near genes of the complement system are associated with altered concentrations of complement protein components in the systemic circulation of AMD patients. The AMD-protective p.Leu9His (rs4151667) variant in the *CFB* gene is associated with reduced factor B (FB) levels [25,26], while the AMD-risk variant p.Gly119Arg (rs141853578) in the *CFI* gene is associated with reduced factor I (FI) levels [27]. The AMD-risk variant p.Pro167Ser (rs34882957) in the *C9* gene was initially associated with elevated C9 levels [28], but a more recent study rather reported decreased C9 levels in carriers of the p.Pro167Ser variant [29]. Common variants at the *CFH* locus were recently shown to be strongly associated with levels of the factor H-related proteins FHR-1, FHR-2, FHR-3 and FHR-4, which are encoded by the *CFHR* genes located at the *CFH* locus downstream of the *CFH* gene. The AMD risk-conferring variant rs570618[T] is associated with increased FHR-1, FHR-2, FHR-3 and FHR-4 levels, while the AMD-protective variant rs10922109[A] is associated with decreased FHR-1, FHR-2, FHR-3 and FHR-4 levels [30,31,32]. Nevertheless, for many of the AMD-associated genetic variants in or near genes of the complement system, the effect on complement protein levels remains unknown.

A recent study observed strong associations of systemic complement activation measurements (defined by the C3d/C3 ratio) with AMD-associated metabolites, including lipoprotein subfractions (large and very large high-density lipoproteins (L- and XL-HDL), very-low-density lipoproteins (VLDL)), other lipids/apolipoproteins (remnant-C, apolipoprotein B (ApoB), triglycerides), fatty acids (monounsaturated fatty acids (MUFA), saturated fatty acids (SFA), total fatty acids (TotFA)), and amino acids (leucine (Leu), isoleucine (Ile) and alanine (Ala)) [33]. Increased L-and XL-HDL levels were associated with increased complement activation, and both HDL levels and complement activation were increased in AMD patients compared to controls. On the other hand, decreased VLDL, remnant-C, ApoB, triglycerides, MUFA, SFA, TotFA, Ile, Leu and Ala levels were associated with increased complement activation, and these metabolites were decreased in AMD compared to controls. These associations may indicate biological interactions between the main AMD disease pathways: lipid metabolism and complement activation. Associations between other complement components and AMD-associated metabolites have not yet been studied in the context of AMD.

In this study, we performed a comprehensive analysis of 32 complement proteins in plasma samples of AMD patients and controls using mass spectrometry-based semi-quantitative multiplex profiling [34]. First, we aimed to determine associations of complement proteins with demographic factors and with AMD. Next, we aimed to determine the association of genetic variants with levels of complement proteins in plasma. Finally, we aimed to determine associations between AMD-associated metabolites and complement proteins in plasma.

## 2. Results

Using a multiplex selected reaction monitoring (SRM) liquid chromatography–mass spectrometry (MS) assay [34], levels of 64 peptides from 32 proteins of the complement system were measured in 476 plasma samples (details of the measured peptides are provided in Supplementary Table S1). These samples belonged to 221 control individuals and 255 AMD patients selected from the EUGENDA database (descriptive statistics of the study cohort are provided in Supplementary Table S2). Genotyping and metabolomics data was available for the majority of the included individuals.

### 2.1. Complement Peptide Levels Are Associated with Age, Sex, and BMI

First, we evaluated whether complement peptide levels vary with age, sex, smoking and BMI. Pearson correlation analyses were performed on the entire cohort using rank-based inverse normal transformed peptide levels. After correcting for the false discovery rate (FDR), 12 peptides were significantly correlated with age (Table 1, Supplementary Table S3). Peptides originating from factor D (FD), C9 and C7 were positively correlated with age, whereas peptides originating from MASP1/3, FCN3, MASP1, C8G (two peptides), C8A (two peptides), C4BPA and clusterin were negatively correlated with age (Table 1). The most significant correlation was found for factor D (*r* = 0.25, *p*-value = 2.50 × 10^−8^, *P*_FDR =_ 1.60 × 10^−6^, Table 1).

Pearson correlation identified 11 peptides that were significantly correlated with sex (Table 1, Supplementary Table S4). Peptides originating from C6, C4BPB, IC1, FI, C8A and clusterin were higher in females, while peptides originating from MBL2 and FD were lower in males. The most significant association with sex was noted for peptide 23 originating from the C6 protein (*r* = 0.19, *p*-value = 3.17 × 10^−5^, *P*_FDR_ = 2.03 × 10^−3^). No significant correlations were identified for smoking status with complement peptides (Supplementary Table S5). However, nine peptides, originating from CRP, C3, FH, FI, vitronectin, and FB, were all positively correlated with increased BMI (Table 1, Supplementary Table S6). The most significantly BMI-associated peptide originated from the CRP protein (*r* = 0.28, *p*-value = 9.49 × 10^−9^, *P*_FDR_ = 6.07 × 10^−7^). All correlation coefficients were between the absolute value of 0 and 0.3, which suggests weak correlations. A schematic overview of correlations between complement peptide levels and demographics is provided in Supplementary Figure S1.

### 2.2. Association Analysis of Complement Peptide Levels with AMD

**Figure 1:**
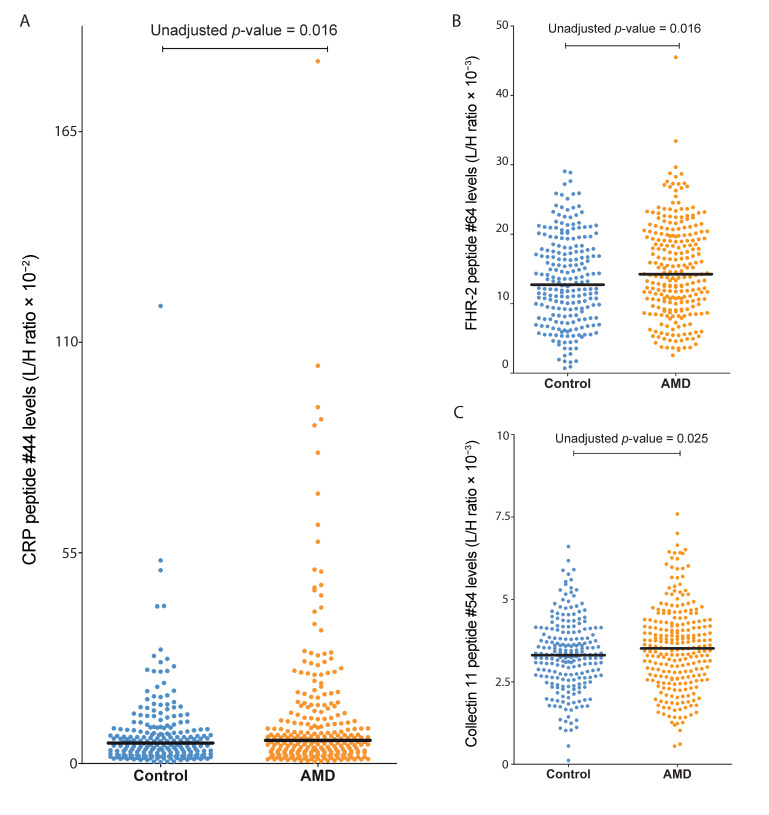
Suggestive association of CRP, FHR-2 and collectin-11 peptide levels with AMD. Showing the distribution of peptide levels (in L/H ratio = endogenous peptide/heavy labelled standard) in blood plasma from controls and AMD patients for (**A**) CRP peptide #44 levels (ESDTSYVSLK), (**B**) FHR-2 peptide #64 levels (TGDIVEFVCK), and (**C**) collectin-11 peptide #54 levels (VFIGINDLEK). *p*-values represent the results from the regression model that is adjusted for age, gender, BMI, smoking status and triglyceride levels.

To determine associations of complement peptide levels with AMD, we performed an association analysis of rank-based inverse normal transformed peptide levels with AMD status by means of linear regression analysis adjusted for age, sex, BMI, smoking and triglycerides, since triglyceride levels were previously associated with complement activation levels [35]. After correction for multiple testing using FDR, no peptides remained significantly associated with AMD status (Supplementary Table S7). However, peptides originating from CRP, FHR-2 and collectin-11 had a *p*-value < 0.05 prior to FDR correction (Table 2) and they were elevated in AMD patients compared to controls (Figure 1A–C).

### 2.3. Complement Peptide Levels Are Associated with AMD Variants

Next, we aimed to determine associations of complement peptide levels with AMD-associated genetic variants. The list of all the variants included in our study can be found in Supplementary Table S7. We initially focused on cis-acting effects of the genetic variants on complement peptide levels, thus we studied associations of genetic variants with proteins encoded by genes located in or near these AMD loci (Table 3, Supplementary Tables S9–S19). Association analyses of complement peptides with AMD-associated variants as identified by the IAMDGC GWAS [6] were carried out adjusting for age, sex and AMD status.

#### 2.3.1. FHR-2 Peptide Levels Are Associated with AMD Variants at the CFH Locus

The multiplex complement assay contains three peptides from FH and one peptide from FHR-2, which are both encoded by genes located at the *CFH* locus (Supplementary Table S1). Eight genetic variants at the *CFH* locus were previously associated with AMD (rs570618, rs121913059, rs187328863, rs35292876, rs191281603, rs10922109, rs148553336 and rs61818925) [6]. Association analyses were performed for these eight variants with FH and FHR-2 peptide levels. After correction for multiple testing using FDR, no peptides from FH were significantly associated with genetic variants at the *CFH* locus, while strong associations were identified for the FHR-2 peptide with rs61818925, rs570618, rs10922109 and rs148553336 (Table 3, Supplementary Tables S9 and S10).

**Figure 2:**
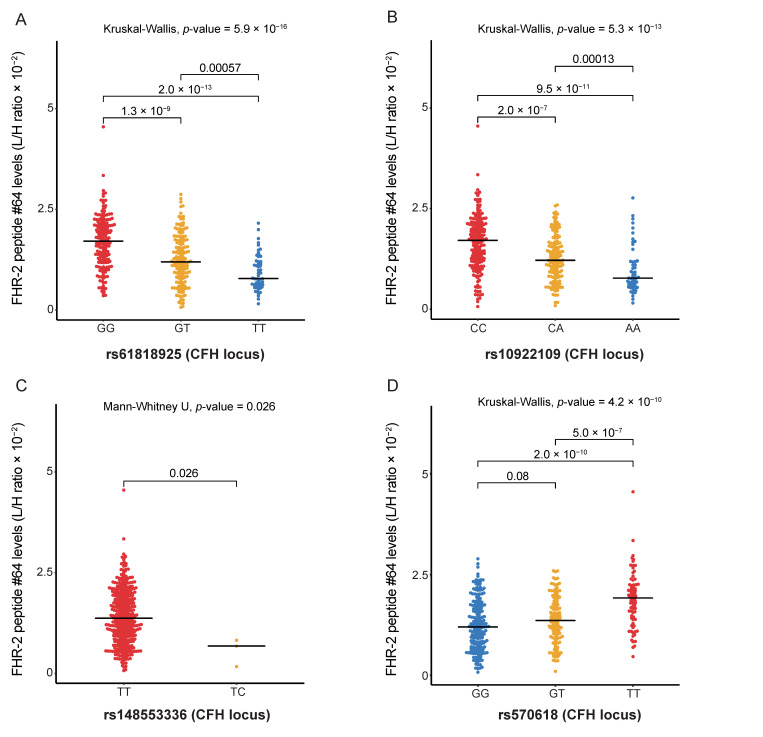
Factor-H related-2 (FHR-2) peptide levels show significant differences between genotype groups of AMD-associated variants at the CFH locus. Red indicates homozygous AMD-risk increasing genotype, while blue shows the homozygous AMD protective genotype and yellow indicates the heterozygous genotype. The Kruskal–Wallis test was included to test if there was a difference between the three genotype groups, where possible. Medians of two genotype groups were compared with the Mann–Whitney-U test. Showing the distribution of peptide levels in light/heavy (L/H) ratio in blood plasma for (**A**) FHR-2 peptide #64 (TGDIVEFVCK), stratified by rs61818925 genotype at the CFH locus; (**B**) FHR-2 peptide #64 (TGDIVEFVCK), stratified by rs10922109 genotype at the CFH locus; (**C**) FHR-2 peptide #64 (TGDIVEFVCK), stratified by rs148553336 genotype at the CFH locus; (**D**) FHR-2 peptide #64 (TGDIVEFVCK), stratified by rs570618 genotype at the CFH locus.

The AMD variant rs61818925 was strongly associated with higher FHR-2 peptide levels (B = 0.570, SE = 0.075, *p*-value = 2.84 × 10^−13^, *P*_FDR_ = 1.13 × 10^−12^, Table 3). The median FHR-2 peptide levels were significantly lower in individuals carrying the TT genotype compared to those carrying the GG genotype for rs61818925 (*p*-value = 2.0 × 10^−13^, Figure 2A). The AMD variant rs10922109 was associated with higher FHR-2 peptide levels (B = 0.540, SE = 0.078, *p*-value = 2.20 × 10^−11^, *P*_FDR_ = 5.13 × 10^−11^). The median FHR-2 peptide levels were significantly lower in individuals carrying the homozygous AA genotype compared to those carrying the homozygous CC genotype for rs10922109 (*p*-value = 9.5 × 10^−11^, Figure 2B). The AMD variant rs148553336 was associated with higher FHR-2 peptide levels (B = 1.320, SE = 0.585, *p*-value = 0.025, *P*_FDR_ = 0.04), with significantly lower median FHR-2 peptide levels in individuals carrying the minor TC genotype compared to those carrying the homozygous TT genotype for rs148553336 (*p*-value = 0.026, Figure 2C). The AMD variant rs570618 was associated with higher FHR-2 peptide levels (B = 0.560, SE = 0.074, *p*-value = 3.24 × 10^−13^, *P*_FDR_ = 1.13 × 10^−12^). Individuals carrying the homozygous GG genotype had significantly lower median FHR-2 peptide levels compared to those carrying the homozygous TT genotype for rs570618 (*p*-value = 2.0 × 10^−10^, Figure 2D).

Associations of peptides from FH with genetic variants at the *CFH* locus did not reach significance after FDR correction, but three variants showed suggestive associations with FH peptide levels with a *p*-value <0.05 prior to FDR correction (Table 3; Supplementary Table S10). The median FH level comparisons with *CFH* locus variants showed borderline significance between different genotypes and can be seen in Supplementary Figure S2A–C.

In addition to the eight AMD-associated variants at the *CFH* locus, a collective enrichment of rare coding variants in the *CFH* gene has been reported in AMD using gene-based approaches [6]. Two rare *CFH* variants were present in this study cohort in more than one individual: *CFH* p.Arg175Gln and *CFH* p.Ser193Leu (details of the number of carriers for rare variants are displayed in Supplementary Table S11). A Mann–Whitney U test was performed comparing FH levels in carriers versus non-carriers. Both coding variants in the *CFH* gene were not associated with altered FH peptide levels.

#### 2.3.2. C4 Peptide Levels Are Associated with AMD Variants at the C2/CFB/SKIV2L Locus

**Figure 3:**
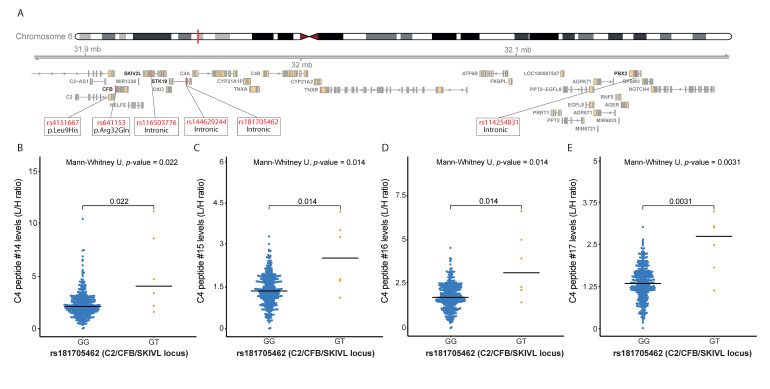
Complement C4 peptide levels show significant differences between genotype groups of AMD-associated variants at the C2/CFB/SKIV2L locus. Blue shows the homozygous AMD protective genotype and yellow indicates the heterozygous genotype. Medians of two genotype groups were compared with the Mann–Whitney-U test. (**A**) Overview of the C2/CFB/SKIV2L locus, showing the four top AMD-associated variants at this locus and two coding variants in CFB tagged by rs116503776, indicating their location in relation to the genes at this locus. Graphs showing the distribution of peptide levels in light/heavy (L/H) in blood plasma for (**B**) complement C4 peptide #14 (DFALLSLQVPLK), stratified by rs181705462 genotype at the C2/CFB/SKIV2L locus; (**C**) complement C4 peptide #15 (VDFTLSSER), stratified by rs181705462 genotype at the C2/CFB/SKIV2L locus; (**D**) complement C4 peptide #16 (VGDTLNLNLR), stratified by rs181705462 genotype at the C2/CFB/SKIV2L locus; (**E**) complement C4 peptide #17 (SHALQLNNR), stratified by rs181705462 genotype at the C2/CFB/SKIV2L locus.

Four genetic variants are independently associated with AMD at the *C2*/*CFB*/*SKIV2L* locus [6], which encompasses four complement genes: the *C2*, *CFB*, *C4A* and *C4B* genes. Notably, the rs181705462 variant is located closer to the *C4A* gene (3 kb) than to the *C2* (34 kb) and *CFB* (27 kb) genes (Figure 3A). The multiplex complement assay contains three peptides from FB, two peptides from C2, and four peptides from C4 (Supplementary Table S1). Association analyses were performed for the four AMD-associated variants at the *C2*/*CFB*/*SKIV2L* locus with FB, C2 and C4 peptide levels. After correction for multiple testing using FDR, no peptides from FB and C2 were associated with genetic variants at the *C2*/*CFB*/*SKIV2L* locus (Supplementary Tables S12 and S13), while significant associations were identified for all four C4 peptides with the rs181705462 variant located near the *C4A* gene (Table 3, Supplementary Table S14). The AMD variant rs181705462 was associated with higher C4 peptide levels for all four C4 peptides, of which C4 peptide 17 showed the strongest effect (B = 1.713, SE = 0.401, *p*-value = 2.55 × 10^−5^, *P*_FDR_ = 0.001). The median C4 peptide levels were significantly higher in individuals carrying the GT genotype compared to those carrying the homozygous GG genotype for rs181705462 (*p*-value = 0.0031 for peptide 17; Figure 3B–E).

The rs116503776 variant at the *C2*/*CFB*/*SKIV2L* locus tags two coding variants in the *CFB* gene: p.Leu9His (rs4151667) and p.Arg32Gln (rs641153) [6] (Figure 3A). The p.Leu9His (rs4151667) variant was previously associated with reduced FB levels [25,26]. We therefore also analysed the median levels of FB in relation to the p.Leu9His (rs4151667) genotype, however, no significant difference was observed. Supplementary Figure S3A–C).

#### 2.3.3. VTN Peptide Levels Are Associated with AMD Variants at the TMEM97/VTN Locus

**Figure 4:**
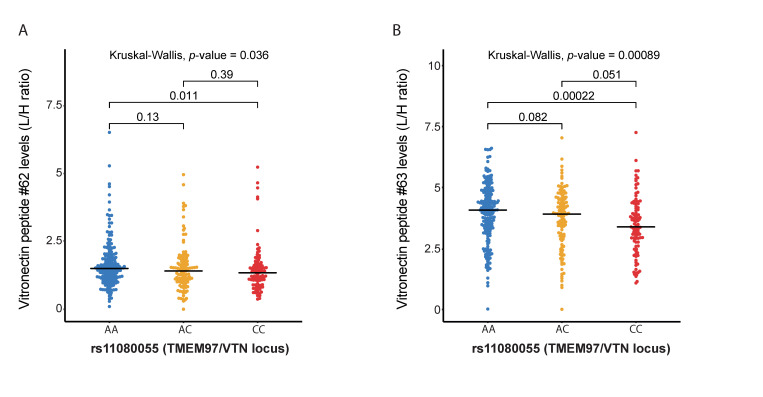
Vitronectin (VTN) levels show significant differences between genotype groups of AMD-associated variants at the TMEM97/VTN locus. Red indicates homozygous AMD-risk increasing genotype, while blue shows the homozygous AMD protective genotype, and yellow indicates the heterozygous genotype. The Kruskal–Wallis test was included to test if there was a difference between the three genotype groups, where possible. Medians of two genotype groups were compared with the Mann–Whitney-U test. Showing the distribution of peptide levels in light/heavy (L/H) in blood plasma for (**A**) VTN peptide #62 (DVWGIEGPIDAAFTR), stratified by rs11080055 genotype at the C2/CFB/SKIV2L locus; (**B**) VTN peptide #63 (FEDGVLDPDYPR), stratified by rs11080055 genotype at the C2/CFB/SKIV2L locus.

The multiplex complement assay contains two peptides originating from vitronectin (VTN). A common variant (rs11080055) at the *TMEM97*/*VTN* locus is independently associated with AMD [6]. The rs11080055 variant was associated with higher VTN peptide levels (for peptide 63: B = 0.206, SE = 0.072, *p*-value = 0.005, *P*_FDR_ = 0.010; Table 3, Supplementary Table S15). The median VTN peptide levels were lower in individuals carrying the homozygous CC genotype compared to those carrying the homozygous AA genotype for rs11080055 (for peptide 63: *p*-value = 0.00022; Figure 4A,B).

#### 2.3.4. FI Peptide Levels Are Associated with AMD Variants at the CFI Locus

Two genetic variants at the *CFI* locus are associated with AMD: the common variant rs10033900 and the rare coding variant p.Gly119Arg (rs141853578) [6]. The target peptide for FI encompasses the p.Gly119 residue, thus the multiplex complement assay cannot be used to reliably analyse the effect of the p.Gly119Arg (rs141853578) variant on FI levels. The AMD risk-conferring variant rs10033900 was not significantly associated with FI peptide levels (Table 3, Supplementary Table S16). In addition to the two AMD-associated variants, a collective enrichment of rare coding variants in the *CFI* gene has been reported in AMD using gene-based approaches [6,16]. The following variants were present in this study cohort in more than one individual: *CFI* p.Leu131Arg, *CFI* p.Arg406His and *CFI* p.Pro553Ser (details of the number of carriers for rare variants are displayed in Supplementary Table S11). A Mann–Whitney U test was performed comparing carriers versus non-carriers for both rare and common variants which suggested significant difference *CFI* p.Leu131Arg variant (Supplementary Figure S4).

#### 2.3.5. C9 Peptide Levels Are Associated with AMD Variants at the C9 Locus

**Figure 5:**
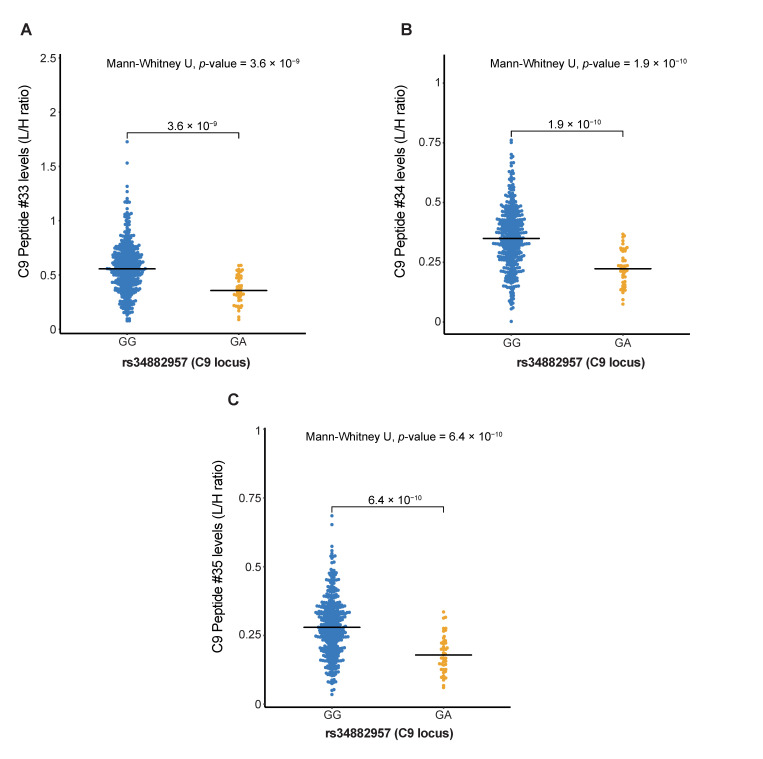
Complement C9 levels show significant differences between genotype groups of rare AMD risk variants at the C9 locus. Showing the distribution of peptide levels in light/heavy (L/H) in blood plasma for (**A**) peptide #33 (TSNFNAAISLK), (**B**) peptide #34 (VVEESELAR) and (**C**) peptide #35 (LSPIYNLVPVK), stratified by C9 p.Pro167Ser (rs34882957) genotype. Blue indicates homozygous AMD protective genotype, and yellow indicates the heterozygous genotype.

A low-frequency coding variant in the *C9* gene, rs62358361, is associated with AMD [6]. The multiplex complement assay contains three peptides originated from C9; however, association analyses that were performed for C9 peptide levels with the *C9* rs62358361 variant showed no significant results (Supplementary Table S17). The coding variant p.Pro167Ser (rs34882957) was previously associated with altered C9 levels [28,29]. The Mann–Whitney U test comparing carriers versus non-carriers identified significant differences in levels of all three C9 peptides with *C9* genotype (*p*-value = 1.91 × 10^−10^ for peptide #34; Supplementary Table S11). C9 peptide levels are significantly lower in carriers of the *C9* p.Pro167Ser (rs34882957) variant compared to non-carriers (Figure 5A–C).

#### 2.3.6. Association Analysis of C3 Peptide Levels with AMD Variants at the C3 Locus

Three variants in or near the *C3* gene were previously associated with AMD [6]. Two peptides from the C3 protein were included in the multiplex complement assay. Association analyses of C3 peptide levels with *C3* genotypes did not identify any significant associations, neither by using general linear models nor with a Mann–Whitney U test (Supplementary Tables S11 and S18).

#### 2.3.7. Association Analysis of Complement Peptide Levels with AMD-Associated Genetic Variants at Other Loci

In addition to cis-acting effects, we also analysed the effects of AMD-associated genetic variants on complement peptide levels originated from proteins encoded by other loci. These associations did not reach statistical significance after FDR correction, but 173 suggestive associations with complement peptide levels with a *p*-value < 0.05 prior to FDR correction were identified (Supplementary Table S19). Within the complement system loci, there were three variants that had more than 10 suggestive associations with other complement protein peptides: the AMD risk-conferring variant rs12019136 at the *C3* locus showed suggestive associations with increased levels of 11 peptides, the protective AMD variant rs148553336 at the *CFH* locus showed suggestive associations with decreased levels of 14 peptides, and the AMD risk-conferring variant rs181705462 at the *C2*/*CFB*/*SKIV2L* locus showed suggestive associations with increased levels of 11 peptides. 

### 2.4. Complement Peptides Are Associated with Lipoproteins and Other AMD Metabolites

In a large metabolomics study including 2267 AMD cases and 4266 controls, a total of 60 metabolites were found to be associated with AMD [33] (see Supplementary Table S20 with the associated metabolites and the effect on AMD). These metabolites included elevated levels of large and extra-large HDL subclasses, decreased levels of VLDL, citrate and amino acids. Moreover, 57 out to these AMD metabolites were associated with complement activation levels measured as the C3d/C3 ratio.

A total of 452 samples (239 cases, 213 controls) from the previous metabolomics study were also included in the current complement profiling study, which allowed us to assess whether complement peptides are associated with AMD metabolites. Association of complement peptide levels with AMD metabolites was performed adjusting for age, sex and AMD status. After FDR adjustment for multiple testing, a total of 327 significant associations were documented (Table 4, Supplementary Table S21). The strongest association was found for a C3 peptide with lower total cholesterol in very large HDL particles (B = −0.250, SE = 0.044, *p*-value = 2.79 × 10^−8^, *P*_FDR_ = 3.70 × 10^−5^), Table 4. The top 20 associations were found for peptides in C3, FH, C9 and vitronectin. These peptides all associated with lower HDL subparticle levels except for C9, which was associated with higher phenylalanine levels (Table 4). These top 20 associations were also observed in a stratified analysis for disease status (Supplementary Table S21).

Of the 327 significantly associated metabolites, those increased in AMD are HDL cholesterol, HDL-2 cholesterol and subparticles of large and extra-large HDL (Supplementary Tables S20 and S21). These HDL-related metabolites were all associated with decreased peptides levels of C3, FH, vitronectin, CRP, FCN3, FI, MASP3, FB, MASP1, C4BPB, C1QA, C8B, C2, FD and properdin (Supplementary Table S21). Metabolites decreased in AMD were VLDL subparticles, apoB, amino acids, citrate, several triglyceride measurements, fatty acid measurements and remnant cholesterol (Supplementary Tables S20 and S21). These metabolites were associated both with higher complement peptide levels and with lower complement peptide levels (Supplementary Table S21). VLDL subparticles were associated with higher peptide levels of FCN3, and lower peptide levels of C7 and C9. APOB was associated with higher levels of FCN3 and lower levels of C7.

The amino acid phenylalanine was associated with higher CRP, FB, C1QA and C8G, isoleucine and leucin were associated with higher FCN3 and alanine was associated with lower C9 and CRP peptide levels. Citrate was associate with lower CRP and C8B peptide levels. Serum triglyceride levels were associated with higher FCN3 levels and lower levels of C7 and C9, and triglyceride in HDL was associated with lower levels of C1QB, C1QC, C1R, C2, C6, C7, C8B, C8G and C9. Fatty acid measurements were associated with higher FCN3 peptide levels, and lower C1QC, C7 and C9 peptide levels. Finally, remnant cholesterol was associated with lower C7 levels (Supplementary Table S21).

## 3. Discussion

**Figure 6:**
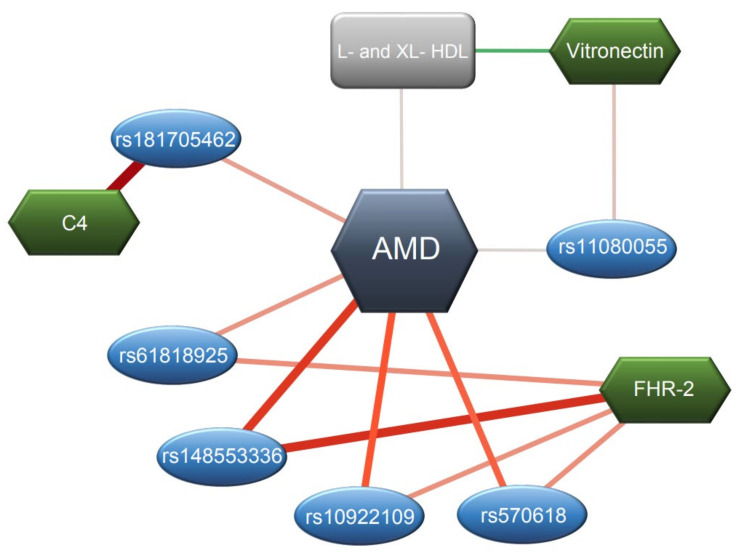
Schematic overview of associations of complement proteins with genetic variants and metabolites. AMD is highlighted in the center in red, complement proteins are highlighted in blue diamonds, genetic variants are highlighted in purple circles and metabolites are highlighted in yellow squares. Only the significantly associated proteins, genetic variants and metabolites are shown. Green colored connections show associations with beta estimates smaller than zero, which indicate associations with lower levels. Red colored connections show associations with beta estimates larger than zero, which generally indicate associations with higher levels. The color of the line is closer to gray as the beta estimate gets closer to zero. Association lines are represented thicker for the higher absolute beta estimates, and thinner for the lower absolute beta estimate.

In this study we performed a comprehensive analysis of 32 complement proteins in plasma samples of AMD patients using mass spectrometry-based semi-quantitative multiplex profiling [34]. We detected significant associations of complement protein levels with age, sex and BMI, and identified potential associations of CRP, FHR-2 and collectin-11 with AMD. In addition, our proteogenomics analyses identified significant associations of genetic variants with levels of complement proteins in plasma, adjusted for AMD status, sex and age: FHR-2 peptide levels were associated with AMD variants at the *CFH* locus, C4 peptide levels were associated with an AMD variant at the *C2*/*CFB*/*SKIV2L* locus, VTN peptide levels were associated with an AMD variant at the *TMEM97*/*VTN* locus, FI peptide levels were associated with AMD variants at the *CFI* locus and C9 peptide levels were associated with an AMD variant at the *C9* locus. Finally, we detected significant associations between AMD-associated metabolites and complement proteins in plasma, also adjusted for AMD status, sex and age. The most significant complement-metabolite associations included HDL subparticle levels with decreased C3, FH and VTN levels. A schematic overview of the identified associations is provided in Figure 6.

We observed an increase of FD, C9 and C7 levels with age, whereas MASP1/3, FCN3, MASP1, C8G, C8A, C4BPA and clusterin decreased with age. A previous study also detected an increase of C9 levels with age [36], which is in agreement with our findings. However, that study reported increased C8 and decreased FD levels with age, which does not correspond to our findings. A potential explanation for the discrepancies between the studies is the difference in age range: the study by Gaya de Costa et al. included a population of healthy individuals with an age range of 20 to 69 years (mean age 45 years) [36], while the AMD case–control cohort described in this study has a mean age of 74 years (Supplementary Table S2). Additional studies specifically in the elderly population will gain a better understanding of the effect of ageing on complement component concentrations across the entire age spectrum. We also identified significant associations of sex and BMI with complement peptide levels. Peptides originating from C6, C4BPB, IC1, FI, C8A and clusterin were increased in females, while peptides originating from MBL2 and FD were decreased. A previous study also detected significant sex differences of complement activity and complement levels in a healthy population [36]. In that study lower concentrations of C3, properdin, MBL, ficolin-3 and terminal component levels were found in females, while FD concentrations were higher. The difference between sex-associated complement proteins could be caused by age difference as it was mentioned for age associations as well. In our current study, peptides originated from CRP, C3, FH, FI, vitronectin and FB, and were all positively correlated with increased BMI. This finding is in agreement with previous studies, which demonstrated that complement factors are expressed in adipose tissue and increased CRP, C3, FH and FB levels are positively associated with BMI [37,38].

None of the analysed levels of complement-derived peptides were significantly associated with AMD after FDR correction. However, the levels of three distinct peptides had a *p*-value < 0.05 prior to correction and are thus suggestive for association with AMD. In our analysis, the peptide from CRP was elevated in AMD patients compared to controls, which confirms previous studies reporting elevated CRP levels in multiple cohorts [39]. In addition, a peptide from the FHR-2 protein was elevated in AMD patients compared to controls, which is in agreement with our recent work reporting elevated FHR-2 levels, in addition to elevated FHR-1, FHR-3 and FHR-4 levels in AMD patients using ELISA measurements [30,31]. Finally, a peptide from collectin-11 was elevated in AMD patients compared to controls. A previous study demonstrated that binding of collectin-11 to retinal pigment epithelial cells induces complement activation [40]. Collectin-11 was shown to activate inflammatory responses through recognition of L-fucose, and fucosidase-treated RPE cells failed to activate complement. This suggests that collectin-11 is of relevance to the inflammatory status of RPE cells and may play a role in AMD pathogenesis. Further analysis of collectin-11 in larger AMD case–control cohorts is warranted to further confirm this association.

This study enabled us to systematically analyse the effect of genetic variants on concentrations of complement components. Our study confirmed previously described associations of FHR-2 levels with genetic variants at the *CFH* locus [31], and reduced FB levels with the p.Leu9His (rs4151667) variant in the *CFB* gene [25,26]. The AMD-risk variant p.Pro167Ser (rs34882957) in the *C9* gene was initially associated with elevated C9 levels [28], but a more recent study rather reported decreased C9 levels in carriers of the p.Pro167Ser variant [29]. The current study identified decreased C9 levels in carriers of the p.Pro167Ser variant in the *C9* gene and gave consistent results for three independent peptides from C9, supporting the latter study that the C9 variant levels are genuinely lower.

Importantly, our study also identified new associations of AMD variants with complement levels. The AMD risk-conferring variant rs181705462 at the *C2*/*CFB*/*SKIV2L* locus was associated with higher C4 peptide levels. The rs181705462 variant is located closer to the *C4A* gene (3 kb) than to the *C2* (34 kb) and *CFB* (27 kb) genes, supporting that the variant may be a relevant determinant of C4 levels, yet the complexity of this locus does not allow us to draw strong conclusions. A previous study by Grassmann et al. reported a protective effect of copy number variation of the *C4A* gene on AMD [41]. The copy number variation was reported to be tagged by rs429608, while the *C4A* copy number variation is independent of rs181705462. This suggests that the elevated C4 peptide levels associated with rs181705462 are not driven by the *C4A* copy number variation. Moreover, the direction of effect in our study seems to be opposite to the study by Grassmann et al., as our study identified an association of increased AMD risk with higher C4 peptide levels, while the study by Grassmann et al. suggests a protective effect with increased *C4A* copy number and thus increased C4A protein levels. Our mass spectrometry-based assay was not able to differentiate between the C4A and C4B isoforms as the targeted peptides are identical in both isoforms (the isoforms differ in only several amino acids). Further studies are needed to determine which isoform is elevated and how this relates to a higher AMD risk conferred by the rs181705462 genotype.

Our study also demonstrates that the AMD variant rs11080055 at the *TMEM97*/*VTN* locus is associated with increased VTN peptide levels. This is in agreement with a recent study that examined the effect of rs704, a non-synonymous variant in the *VTN* gene [42]. The AMD risk-conferring variant rs704 is associated with increased VTN expression, supporting the involvement of altered VTN levels in AMD pathogenesis. VTN is an inhibitor of the terminal complement complex, and also serves various other functions, such as maintaining retinal integrity [42]. The rs704 variant induces collagen accumulation, and VTN is a major component of drusen and subretinal drusenoid deposits. It was also reported in the same study that rs704 causes a change in vitronectin protein, resulting in two isoforms. Our mass spectrometry assay was not designed to differentiate between these isoforms specifically. Therefore, we could not study the differences of these isoforms.

Additionally, we observed a suggestive association of the AMD risk-conferring variant rs10033900 at the *CFI* locus with reduced FI levels. A recent eQTL study reported associations of genetic variants (that are in high linkage disequilibrium with rs10033900) at the *CFI* locus with decreased gene expression of *CFI* in the retina [43]. Reduced FI levels have previously also been reported for the p.Gly119Arg variant and other rare coding variants in the *CFI* gene [27,44]. The effect of the p.Gly119Arg variant on FI levels could not reliably be analysed with the multiplex complement assay used in this study, as the target peptide for FI encompasses the p.Gly119 residue. In this study we confirmed that the rare coding *CFI* variant p.Leu131Arg is associated with lower FI levels, while no effect on FI levels was noted for the p.Arg406His and *CFI* p.Pro553Ser variants [44].

In addition to cis-acting effects, we also identified potential associations of complement peptide levels with genetic variants at other AMD loci, but none of these associations remained significant after FDR correction. Several variants showed suggestive associations with multiple complement components. For example, the protective AMD variant rs148553336[C] at the *CFH* locus showed suggestive associations with decreased FI, C3, FB and VTN peptide levels, in addition to decreased FHR-2 and FH associated with cis-acting effects at the *CFH* locus. The AMD risk-conferring variant rs181705462 at the *C2*/*CFB*/*SKIV2L* locus showed suggestive associations with increased FI, C9, C3 and FH peptide levels, in addition to increased C4 levels. A recent study using the same assay for semi-quantitative multiplex profiling of the complement system in patients with various complement deficiencies also identified potential trans-acting effects of mutations in complement genes [45]. This may be explained by functional coupling of complement factors within the pathways down- or upstream of the affected protein. Semi-quantitative multiplex profiling of the complement system in larger AMD case–control cohorts can further solidify trans-acting effects of genetic variants on a wide range of complement proteins.

The associations between genetic variants and the peptides of complement proteins were further checked in the QTLbase database (http://www.mulinlab.org/qtlbase/index.html, accessed on 12 November 2021) to assess novelty of our findings. In this database, rs570618 was reported to be associated with FH proteins. Only the effect estimates for hemopexin was reported for CFH, which was 0.09 (beta value). Given how small the effect estimate is, we would need higher statistical power to detect it in our study. For the variant rs10922109, an association with hemopexin was shown with the beta estimate of −0.13 within the database. We have shown a similar effect estimate for this variant (−0.16) which lost its statistical significance after multiple test correction. Lastly, the variant rs11080055 was shown as a pQTL; however, the effect estimate is not provided in the database.

Finally, we detected strong associations between metabolites and complement components. A recent study observed strong associations of systemic complement activation measurements (defined by the C3d/C3 ratio) with AMD-associated metabolites, including HDL and VLDL lipoprotein subfractions, other lipids/apolipoproteins (remnant-C, ApoB and triglycerides), fatty acids (MUFA, SFA and TotFA) and amino acids (Leu, Ile and Ala) [33]. In the current study, we extended these associations to a broad range of metabolites and complement components. Increased HDL levels were previously associated with an increased risk of AMD [33,46], and in this study we show that increased HDL levels are associated with decreased C3, FH, VTN, CRP, FCN3, FI, MASP3, FB, MASP1, C4BPB, C1QA, C8B, C2 and FD and properdin levels. Increased HDL levels have previously been associated with decreased FH and FB levels [37]; our study thus confirms and further extends these findings. In addition, our current study identified strong associations of VLDL subparticles, amino acids, citrate and fatty acids with altered complement component levels. Strong associations between AMD-associated metabolites and complement components may indicate biological interactions between the main AMD pathways, including the lipid metabolism and the complement system.

The results of this study have several implications for clinical translation. Multiple clinical trials, either targeting the central components C5 and C3 or complement regulators, have been completed or are currently ongoing [47]. Most of the candidate therapeutic complement compounds that have been tested so far have shown limited success [48]. Our current study suggests that several factors including age, sex, BMI, genetic variants and circulating metabolites may affect complement protein concentrations and may thus influence treatment success. The observed age-related differences of concentrations of complement components suggest that age should be considered in the design of therapeutics targeting the complement system, as different doses may be required among different age groups [36]. In addition, genetic variants may be considered for patient inclusion in clinical trials to increase the efficacy of the treatments that are being tested. Variants at the *CFH*, *CFI*, *C2*/*CFB*, *C9* and *VTN* loci are associated with altered complement component concentrations and could therefore be used to select patients for compounds targeting specific complement components. Two clinical trials supplementing FH (GEM103, Gemini Therapeutics) and *CFI* (GT005, Gyroscope Therapeutics) are the first to be selecting patients based on genotype (carrying risk variants in *CFH* and *CFI*, respectively) before inclusion in the trials. Our current study and previous work by others [17,25,26,29,30,44] suggest that genetic variants at the *CFH*, *CFI*, *C2*/*CFB*, *C9* and *VTN* loci can also be used to design personalised medicine approaches for AMD. Furthermore, the strong associations between circulating metabolites and complement components suggest that multiple disease pathways may need to be targeted for successful treatment of AMD.

Our study has several strengths and weaknesses. A strength of our study is that our semi-quantitative multiplex profiling assay is able to measure a broad range of complement components using a small sample volume [34]. The availability of detailed genetic data and metabolite measurements for our cohort enabled the integration of complement concentrations with various levels of data. Our current study included a fairly large sample size, consisting of 476 plasma samples. Although our sample size was sufficiently large to identify numerous significant associations, even larger studies are needed to follow-up on our identified suggestive associations that remained under the multiple testing adjusted threshold. Moreover, the genetic variants with low MAF (<5%) can be chance based findings due to their rarity and should not be considered to be conclusive with the number of samples included in this study. A limitation of the current design of our multiplex assay is that, due to peptide target design and the detection limit of our mass spectrometry assay, not all complement components can be assessed: FHR-2 could be analysed using the assay, but the other FHR proteins were below the detection limit. In addition, the target peptide for FI is specific for the p.Gly119 residue and can therefore not detect the effect of the *CFI* variant p.Gly119Arg on FI levels. Furthermore, the current assay cannot differentiate between the C4A and C4B isoforms, and further studies are needed to clarify which isoform (or whether the ratio between isoforms) is associated with the rs181705462 genotype. Finally, the current assay has been developed to measure complement component levels and does not allow the measurement of complement activation products.

In conclusion, we performed a comprehensive analysis of 32 complement proteins in plasma samples of AMD patients and controls using semi-quantitative multiplex profiling. Our study did not identify significantly associated peptides with AMD status; however, we identified numerous associations of complement components with age, sex, BMI, genetic variants and circulating metabolites. The results of our study suggest that these factors should be taken into account to design personalized treatment approaches and to increase the success of clinical trials targeting the complement system.

## 4. Materials and Methods

### 4.1. The Study Samples

For this study, EDTA plasma samples from 255 AMD patients and 221 control subjects were selected from the Dutch and German European Genetic Database (EUGENDA-Nijmegen and EUGENDA-Cologne) [49]. These EDTA plasma have been collected and stored (–80 °C within 1 h) according to the standard protocols. All participants provided written informed consent for clinical examination, epidemiological data collection and blood sampling for biochemical and genetic analyses. All studies were approved by the appropriate ethical committees (Commissie Mensgebonden Onderzoek [CMO] Arnhem-Nijmegen for EUGENDA-Nijmegen, Ethics Commission of Cologne University’s Faculty of Medicine for EUGENDA-Cologne). Inclusion/exclusion criteria was based on the completeness of the dataset in terms of AMD grading, age > 55, and genetics and metabolomics data availability. Demographic factors of the study cohort are provided in Supplementary Table S2.

### 4.2. Genotyping

All individuals included in this study had previously been genotyped with a custom-modified Illumina HumanCoreExome array at the Centre for Inherited Disease Research (CIDR) and analysed within the IAMDGC GWAS (43,566 subjects; 16,144 advanced AMD cases and 17,832 controls of European ancestry in the primary analysis dataset) [6].

### 4.3. Metabolomic Analysis

Available metabolomic data from these patients were obtained from a previous study by Acar et al. [33]. In summary, the plasma samples were analysed by means of a high-throughput proton nuclear magnetic resonance (NMR) metabolomics platform (Nightingale Health, Ltd., Helsinki, Finland). After quality control, in total 146 high quality metabolites were selected, including amino acids, glycolysis measures, ketone bodies, inflammation-related measurements, fatty acids, and lipoprotein subclasses (Supplementary Table S20). Univariate logistic regression models were adjusted for age and sex, and were further combined into a random effects meta-analysis to determine the final association estimates.

### 4.4. Complement Targeted Multiplex Assay

Sample preparation and mass spectrometric analysis was performed as previously described [37]. Briefly, the plasma samples were reduced, alkylated, digested and stored at −80 °C. In prior to analysis, the digests were spiked with a mix of C-terminally ^13^C^15^N stable isotope labelled peptide standards (Thermo, JPT) for quantitation (L/H ratio = endogenous peptide/heavy labelled standard) and were desalted using Bond Elut OMIX tips (Agilent). Samples were analysed in 1-min target windows using the Waters Aquity MClass UPLC Xevo TQ-S, equipped with an ESI source and an iKeyTM (Waters peptide BEH C18, 130 Å, 1.7 µm, 150 µm × 100 mm). The peptides were eluted from the column using a gradient from 3 to 35% acetonitrile in 0.1% formic acid in 20 min at a flow rate of 2 µL/min. Raw data were analysed and exported using Skyline software v3.7 (MacCoss Lab, University of Washington, DC, USA [50]). The quality of the dataset was manually inspected to ensure correct peak detection and integration. Peaks that failed manual evaluation had poor technical quality indicators such as <0.75 dot product (ratio of the transitions as compared to the human plasma spectral library (Human_plasma_2012-08_all.splib.zip, build by H. Lam (2012), available at the PeptideAtlas website: http://www.peptideatlas.org/speclib/, last accessed 20 June 2020) or <3× signal-to-noise ratio. They were excluded and reported as ‘invalid’ or ‘below limit of detection’ (<LOD), respectively. This resulted in 64 high quality peptides targets for 32 different complement proteins (Supplementary Table S1).

### 4.5. Statistical Analysis

Statistical analyses were performed using SPSS (version 16.0; IBM) and standard build-in packages in R (version 3.6.3). Pearson correlations were calculated for demographic factors with Hmisc package in R, using Pearson parameter in rcorr function. Rank-based inverse normal transformation was used for the peptide levels. Univariate association analyses with the complement proteins as outcomes were performed using linear regression models adjusted for sex, age, smoking, BMI, and triglyceride levels. A post hoc multiple testing correction was performed to control the false discovery rate (FDR) using the Benjamini–Hochberg procedure to take the high correlation between complement peptides into account (Supplementary Figure S1). Genotype and metabolite associations were performed by univariate linear regression models adjusted by age, sex, and AMD status, with complement proteins as outcomes. Genotype associations were performed to determine the AMD risk increasing alleles. Differences between different genotype groups were explored using the Mann–Whitney U test, and Kruskal–Wallis test where possible. The threshold for statistical significance was defined as an FDR-corrected *p*-value (P_FDR_) of less than 0.05. The *p*-values that were lower than 0.05, but were higher than 0.05 for P_FDR_, were defined as suggestive associations.

## Figures and Tables

**Table 1:** Association of demographic factors with complement peptide levels. Pearson correlation was computed for each demographic factor, and the significantly associated protein peptides are shown. Full list of Pearson correlations to age, sex, smoking and BMI can be found in Supplementary Tables S3–S6.

Demographic	Peptide ID	Protein	Pearson’s *r*	*p*-Value	*p*-Value_FDR_
Age	39	FD	0.25	2.50 × 10^−8^	1.60 × 10^−6^
	46	MASP1/3	−0.19	2.78 × 10^−5^	8.89 × 10^−4^
	52	FCN3	−0.18	9.94 × 10^−5^	2.12 × 10^−3^
	35	C9	0.14	1.65 × 10^−3^	2.64 × 10^−2^
	32	C8G	−0.14	2.80 × 10^−3^	2.94 × 10^−2^
	31	C8G	−0.14	2.85 × 10^−3^	2.94 × 10^−2^
	48	MASP1	−0.13	3.21 × 10^−3^	2.94 × 10^−2^
	25	C7	0.13	3.87 × 10^−3^	3.10 × 10^−2^
	27	C8A	−0.13	4.94 × 10^−3^	3.16 × 10^−2^
	26	C8A	−0.13	4.64 × 10^−3^	3.16 × 10^−2^
	55	C4BPA	−0.12	7.62 × 10^−3^	4.07 × 10^−2^
	60	Clusterin	−0.12	7.07 × 10^−3^	4.07 × 10^−2^
Sex	23	C6	0.19	3.17 × 10^−5^	2.03 × 10^−3^
	50	MBL2	−0.18	9.60 × 10^−5^	3.07 × 10^−3^
	51	MBL2	−0.17	2.06 × 10^−4^	3.29 × 10^−3^
	57	C4BPB	0.17	1.69 × 10^−4^	3.29 × 10^−3^
	58	IC1	0.16	6.38 × 10^−4^	8.17 × 10^−3^
	59	IC1	0.15	7.97 × 10^−4^	8.50 × 10^−3^
	43	FI	0.14	2.61 × 10^−3^	1.86 × 10^−2^
	61	Clusterin	0.14	2.47 × 10^−3^	1.86 × 10^−2^
	60	Clusterin	0.14	2.58 × 10^−3^	1.86 × 10^−2^
	39	FD	−0.14	2.95 × 10^−3^	1.89 × 10^−2^
	27	C8A	0.13	5.85 × 10^−3^	3.40 × 10^−2^
BMI	44	CRP	0.28	9.49 × 10^−9^	6.07 × 10^−7^
	12	C3	0.24	1.20 × 10^−6^	3.85 × 10^−5^
	41	FH	0.21	1.05 × 10^−5^	2.25 × 10^−4^
	43	FI	0.20	5.55 × 10^−5^	8.88 × 10^−4^
	42	FH	0.19	1.27 × 10^−4^	1.63 × 10^−3^
	40	FH	0.18	1.77 × 10^−4^	1.89 × 10^−3^
	63	Vitronectin	0.17	4.03 × 10^−4^	3.69 × 10^−3^
	13	C3	0.14	5.40 × 10^−3^	4.32 × 10^−2^
	38	FB	0.13	6.53 × 10^−3^	4.64 × 10^−2^

**Table 2:** Suggestive associations of complement peptide levels with age-related macular degeneration. Beta values show the effect estimates from the regression analysis results. AMD status was the dependent variable in the regression model, while peptide levels, age, sex, BMI, smoking status and triglyceride levels were added as independent variables.

Peptide id	Protein	B	SE	*p*-Value	*p*-Value_FDR_
64	FHR-2	0.234	0.094	0.013	0.517
44	CRP	0.226	0.094	0.016	0.517
54	Collectin 11	0.187	0.094	0.046	0.913

**Table 3:** Cis-acting effects of AMD-associated genetic variants on complement peptide levels. Complement protein levels in the locus of each known AMD variant were tested for association with each known AMD variant separately. Alleles were chosen for the risk increasing effect.

Peptide Id	Protein	AMD Variant *	Allele	AF	Locus Gene(s) ^	B	SE	*p*-Value	*p*-Value_FDR_	OR AMD ^#^	*p*-Value AMD ^#^
64	FHR-2	rs61818925	G	0.639	*CFH (CFHR3*/*CFHR1)*	0.570	0.075	2.84 × 10^−13^	1.13 × 10^−12^	1.667	6.0 × 10^−165^
64	FHR-2	rs570618	T	0.442	*CFH*	0.560	0.074	3.24 × 10^−13^	1.13 × 10^−12^	2.380	2.0 × 10^−590^
64	FHR-2	rs10922109	C	0.617	*CFH*	0.540	0.078	2.20 × 10^−11^	5.13 × 10^−11^	2.632	9.6 × 10^−618^
64	FHR-2	rs148553336	T	0.996	*CFH*	1.320	0.585	0.025	0.044	3.448	8.6 × 10^−26^
41	FH	rs148553336	T	0.996	*CFH*	1.669	0.573	0.004	0.084	3.448	8.6 × 10^−26^
40	FH	rs148553336	T	0.996	*CFH*	1.399	0.576	0.016	0.112	3.448	8.6 × 10^−26^
42	FH	rs148553336	T	0.996	*CFH*	1.403	0.581	0.016	0.112	3.448	8.6 × 10^−26^
41	FH	rs61818925	G	0.639	*CFH (CFHR3*/*CFHR1)*	0.179	0.079	0.024	0.126	1.667	6.0 × 10^−165^
42	FH	rs10922109	C	0.617	*CFH*	−0.164	0.082	0.047	0.197	2.632	9.6 × 10^−618^
17	C4	rs181705462	T	0.009	*C2*/*CFB*/*SKIV2L*	1.713	0.401	2.55 × 10^−5^	0.001	1.550	3.1 × 10^−10^
15	C4	rs181705462	T	0.009	*C2*/*CFB*/*SKIV2L*	1.485	0.406	2.91 × 10^−4^	0.003	1.550	3.1 × 10^−10^
16	C4	rs181705462	T	0.009	*C2*/*CFB*/*SKIV2L*	1.468	0.409	3.73 × 10^−4^	0.003	1.550	3.1 × 10^−10^
14	C4	rs181705462	T	0.009	*C2*/*CFB*/*SKIV2L*	1.318	0.410	0.001	0.006	1.550	3.1 × 10^−10^
63	Vitronectin	rs11080055	A	0.522	*TMEM97*/*VTN*	0.206	0.072	0.005	0.010	1.099	1.0 × 10^−8^

AF = Allele frequency, * AMD associated variants from the IAMDGC GWAS on AMD (Fritsche et al., 2015) [6], ^ Nearest genes to the variant, ^#^ Results from the primary IAMDGC GWAS in Fritsche et al., 2015 [6].

**Table 4:** Top 20 associations of complement peptide levels with AMD-associated metabolites. Each AMD-associated metabolite was checked for association with complement protein peptides in univariate regression analyses.

Metabolite	OR_AMD_ *	Peptide Id	Protein	B	SE	*p*-Value	*p*-Value_FDR_
XL-HDL-C	1.082	12	C3	−0.250	0.044	2.79 × 10^−8^	3.70 × 10^−5^
XL-HDL-L	1.102	12	C3	−0.246	0.044	2.81 × 10^−8^	3.70 × 10^−5^
XL-HDL-P	1.105	12	C3	−0.249	0.044	2.89 × 10^−8^	3.70 × 10^−5^
XL-HDL-FC	1.092	12	C3	−0.240	0.044	6.61 × 10^−8^	6.34 × 10^−5^
XL-HDL-C	1.082	42	FH	−0.239	0.044	9.93 × 10^−8^	6.71 × 10^−5^
XL-HDL-P	1.105	42	FH	−0.237	0.044	1.05 × 10^−7^	6.71 × 10^−5^
XL-HDL-FC	1.092	42	FH	−0.233	0.044	1.48 × 10^−7^	7.12 × 10^−5^
XL-HDL-L	1.102	42	FH	−0.233	0.044	1.37 × 10^−7^	7.12 × 10^−5^
XL-HDL-PL	1.123	12	C3	−0.229	0.043	1.89 × 10^−7^	8.06 × 10^−5^
Phe	0.857	34	C9	0.227	0.044	2.97 × 10^−7^	9.97 × 10^−5^
L-HDL-P	1.092	12	C3	−0.218	0.042	2.65 × 10^−7^	9.97 × 10^−5^
L-HDL-CE	1.095	12	C3	−0.219	0.042	3.38 × 10^−7^	9.97 × 10^−5^
L-HDL-FC	1.097	12	C3	−0.224	0.043	3.14 × 10^−7^	9.97 × 10^−5^
L-HDL-C	1.095	12	C3	−0.218	0.042	3.90 × 10^−7^	1.07 × 10^−4^
XL-HDL-P	1.105	63	Vitronectin	−0.225	0.044	5.01 × 10^−7^	1.13 × 10^−4^
XL-HDL-PL	1.123	42	FH	−0.220	0.043	5.29 × 10^−7^	1.13 × 10^−4^
L-HDL-P	1.092	42	FH	−0.213	0.042	4.51 × 10^−7^	1.13 × 10^−4^
L-HDL-CE	1.095	42	FH	−0.214	0.042	5.27 × 10^−7^	1.13 × 10^−4^
L-HDL-FC	1.097	42	FH	−0.218	0.043	5.66 × 10^−7^	1.14 × 10^−4^
L-HDL-C	1.095	42	FH	−0.213	0.042	6.12 × 10^−7^	1.18 × 10^−4^

* Acar et al., 2020 [33].

## Data Availability

The complement peptide analysis Skyline and raw data files are available at the Panorama public repository: https://panoramaweb.org/CS_MRM_AMD.url (ProteomeXchange ID: PDX027689) (last accessed 20 June 2020).

## Supplementary Materials

The following are available online at https://www.mdpi.com/article/10.3390/jpm11121256/s1, Figure S1: Correlation heatmap of demographic factors and peptides from proteins, Figure S2: Factor H (FH) peptide levels show significant differences between genotype groups of AMD-associated variants at the CFH locus, Figure S3: Factor B (FB) peptide levels show significant differences between genotype groups of AMD-associated variants at the C2/CFB/SKIV2L locus, Figure S4: Factor I (FI) levels show significant differences between genotype groups of rs10033900 and p.Leu131Arg rare variant at the CFI locus, Table S1. Peptides of the complement system analysed in the multiplex selected reaction monitoring liquid chromatography–mass spectrometry assay, Table S2. Demographic factors, clinical characteristics and other measurements of the study cohort, Table S3. Pearson correlation analyses of peptides in the complement pathway with age, Table S4. Pearson correlation analyses of peptides in the complement pathway with sex, Table S5. Pearson correlation analyses of peptides in the complement pathway with smoking status, Table S6. Pearson correlation analyses of peptides in the complement pathway with BMI, Table S7. Association of peptides in the complement pathway with age-related macular degeneration, Table S8. List of the genetic variants included in this study, Table S9. Association of FHR-2 peptides with *CFH* variants known to be associated with age-related macular degeneration, Table S10. Association of FH peptides with *CFH* variants known to be associated with age-related macular degeneration, Table S11. Rare variants in complement genes in the study cohort and Mann–Whitney U test results with peptide levels between rare variants carriers and non-carriers, Table S12. Association of FB peptides with variants at the *C2*/*CFB*/*SKIV2L* locus, known to be associated with age-related macular degeneration, Table S13. Association of C2 peptides with variants at the *C2*/*CFB*/*SKIV2L* locus, known to be associated with age-related macular degeneration, Table S14. Association of C4 peptides with variants at the *C2*/*CFB*/*SKIV2L* locus, known to be associated with age-related macular degeneration, Table S15. Association of vitronectin peptides with variants at the *TMEM97*/*VTN* locus, known to be associated with age-related macular degeneration, Table S16. Association of FI peptide with variant at the *CFI* locus, known to be associated with age-related macular degeneration, Table S17. Association of C9 peptides with a rare variant at the *C9* locus, known to be associated with age-related macular degeneration, Table S18. Association of C3 peptides with variants at *C3* locus, known to be associated with age-related macular degeneration, Table S19. Association of peptides from the complement system with variants at other AMD loci, Table S20. Metabolites included in the Nightingale platform and their association with AMD, Table S21. Association of peptides in the complement pathway with AMD-associated metabolites.
