# Supplementary material for: Semi-Quantitative Multiplex Profiling of the Complement System Identifies Associations of Complement Proteins with Genetic Variants and Metabolites in Age-Related Macular Degeneration
Source: J Pers Med. 2021 Nov 25;11(12):1256. doi: 10.3390/jpm11121256 (PMC8705464; doi:10.3390/jpm11121256)

Supplementary figure 2. Factor H (FH) peptide levels show significant differences between genotype groups of AMD-associated variants at the CFH locus. Red indicates homozygous AMD-risk increasing genotype, while blue shows the homozygous AMD protective genotype, and yellow indicates the heterozygous genotype. Kruskal Wallis test was included to test if there was a difference between the three genotype groups, where possible. Medians of two genotype groups were compared with the Mann Whitney-U test. Showing the distribution of peptide levels in light/heavy (L/H) ratio in blood plasma for A) FH peptide #41 (SIDVACHPGYALPK), stratified by rs61818925 genotype at the CFH locus; B) FH peptide #42 (SSNLIILEEHLK), stratified by rs10922109 genotype at the CFH locus; C) FH peptide #41 (SIDVACHPGYALPK), stratified by rs148553336 genotype at the CFH locus.

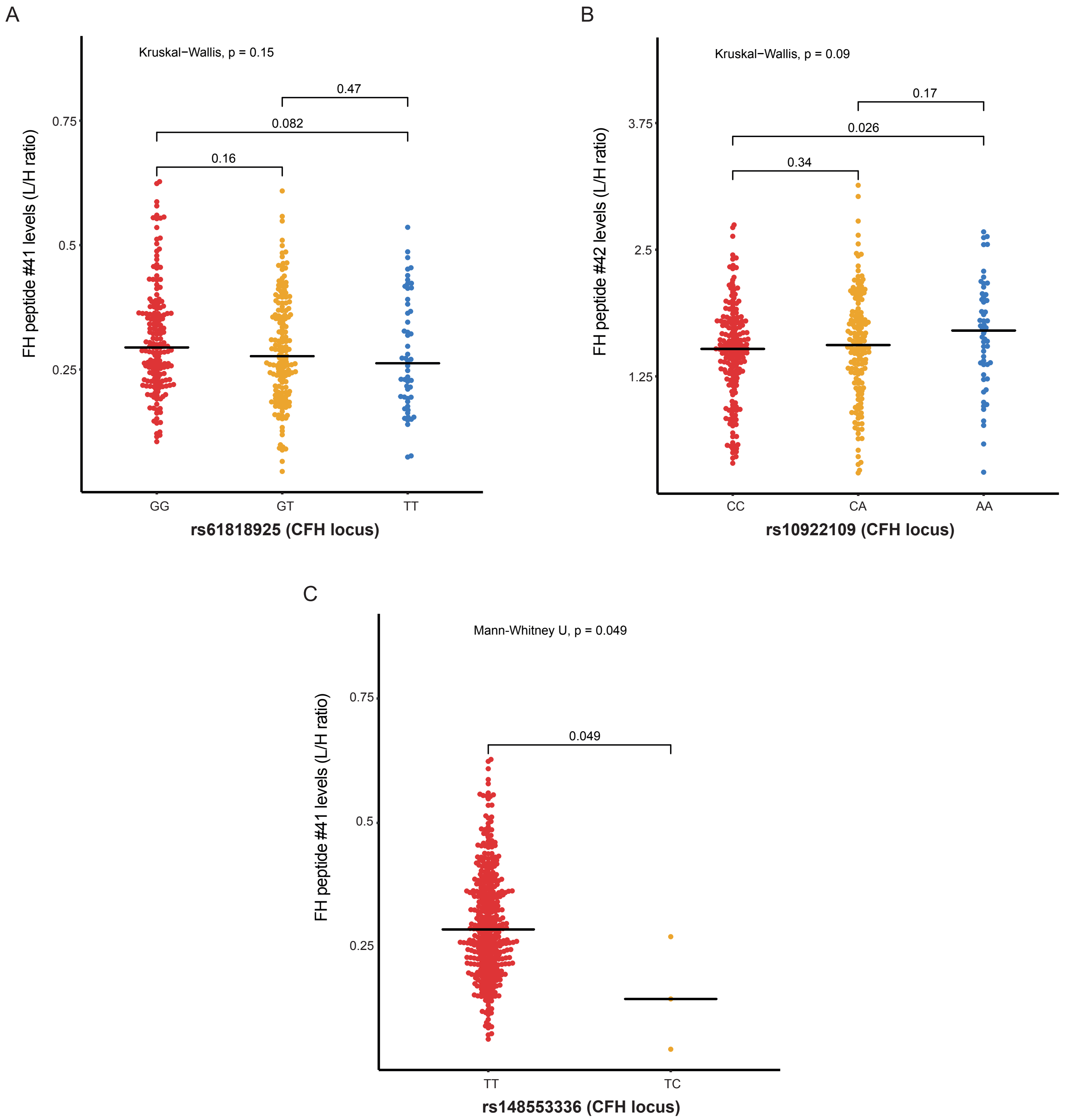

Supplement: Supplementary file 1 [file jpm-11-01256-s001.zip › SupplementaryFigureS2.pdf]
