# Supplementary material for: Semi-Quantitative Multiplex Profiling of the Complement System Identifies Associations of Complement Proteins with Genetic Variants and Metabolites in Age-Related Macular Degeneration
Source: J Pers Med. 2021 Nov 25;11(12):1256. doi: 10.3390/jpm11121256 (PMC8705464; doi:10.3390/jpm11121256)

Supplementary figure 3. Factor B (FB) peptide levels show significant differences between genotype groups of AMD-associated variants at the C2/CFB/SKIV2L locus. Red indicates homozygous AMD-risk increasing genotype and yellow indicates the heterozygous genotype. Medians of two genotype groups were compared with the Mann Whitney-U test. A) FB peptide #36 (STGSWSTLK), stratified by rs4151667 genotype at the C2/CFB/SKIV2L locus; B) FB peptide #37 (YGLVTYATYPK), stratified by rs4151667 genotype at the C2/CFB/SKIV2L locus; C) FB peptide #38 (DAQYAPGYDK), stratified by rs4151667 genotype at the C2/CFB/SKIV2L locus

A

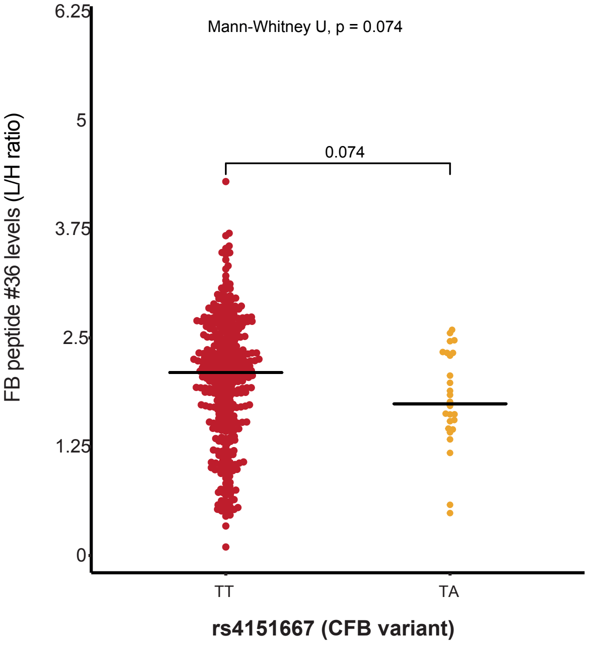

B

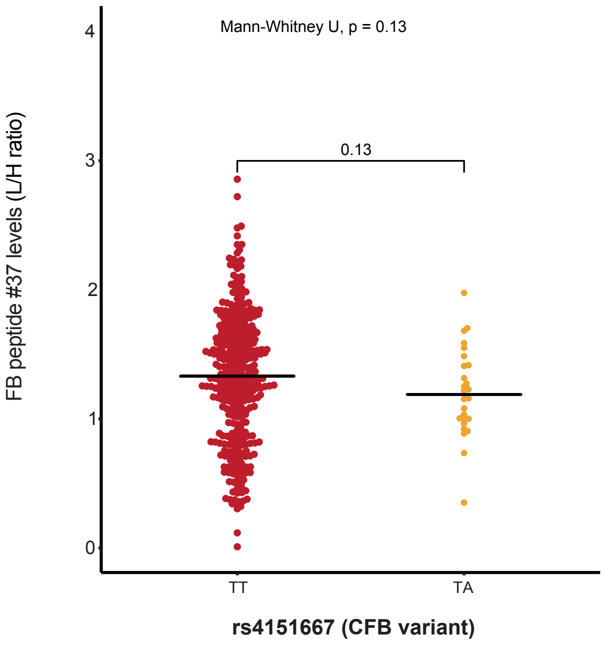

C

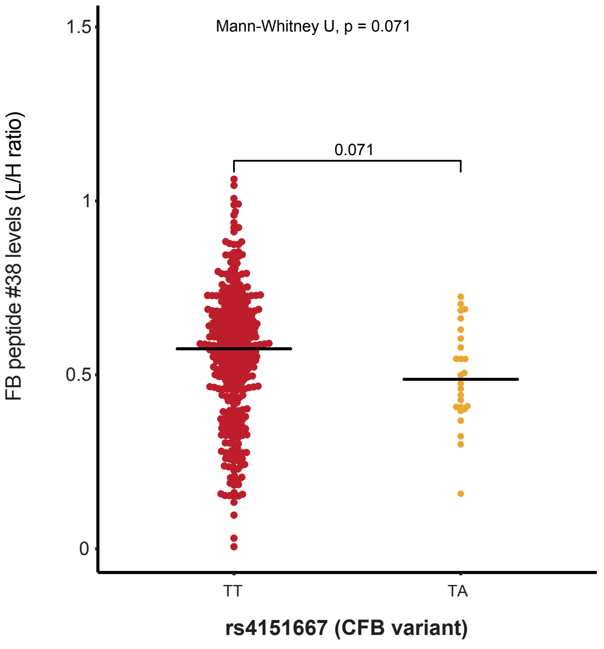

Supplement: Supplementary file 1 [file jpm-11-01256-s001.zip › SupplementaryFigureS3.pdf]
