# Supplementary material for: Semi-Quantitative Multiplex Profiling of the Complement System Identifies Associations of Complement Proteins with Genetic Variants and Metabolites in Age-Related Macular Degeneration
Source: J Pers Med. 2021 Nov 25;11(12):1256. doi: 10.3390/jpm11121256 (PMC8705464; doi:10.3390/jpm11121256)

Supplementary Figure 4. Factor I (FI) levels show significant differences between genotype groups of rs10033900 and p.Leu131Arg rare variant at the CFI locus. Red indicates homozygous AMD-risk increasing genotype, while blue shows the homozygous AMD protective genotype, and yellow indicates the heterozygous genotype. Kruskal Wallis test was included to test if there was a difference between the three genotype groups, where possible. Medians of two genotype groups were compared with the Mann Whitney-U test. Showing the distribution of peptide levels in light/heavy (L/H) in blood plasma for A) FI peptide #43 (HGNTDSEGIVEVK), stratified by rs10033900genotype at the CFI locus; B) FI peptide #43 (HGNTDSEGIVEVK), stratified by CFI p.Leu131Arg genotype; C) FI peptide #43 (HGNTDSEGIVEVK), stratified by CFI p.Arg406His genotype; D) FI peptide #43 (HGNTDSEGIVEVK), stratified by CFI p.Pro553Ser genotype.

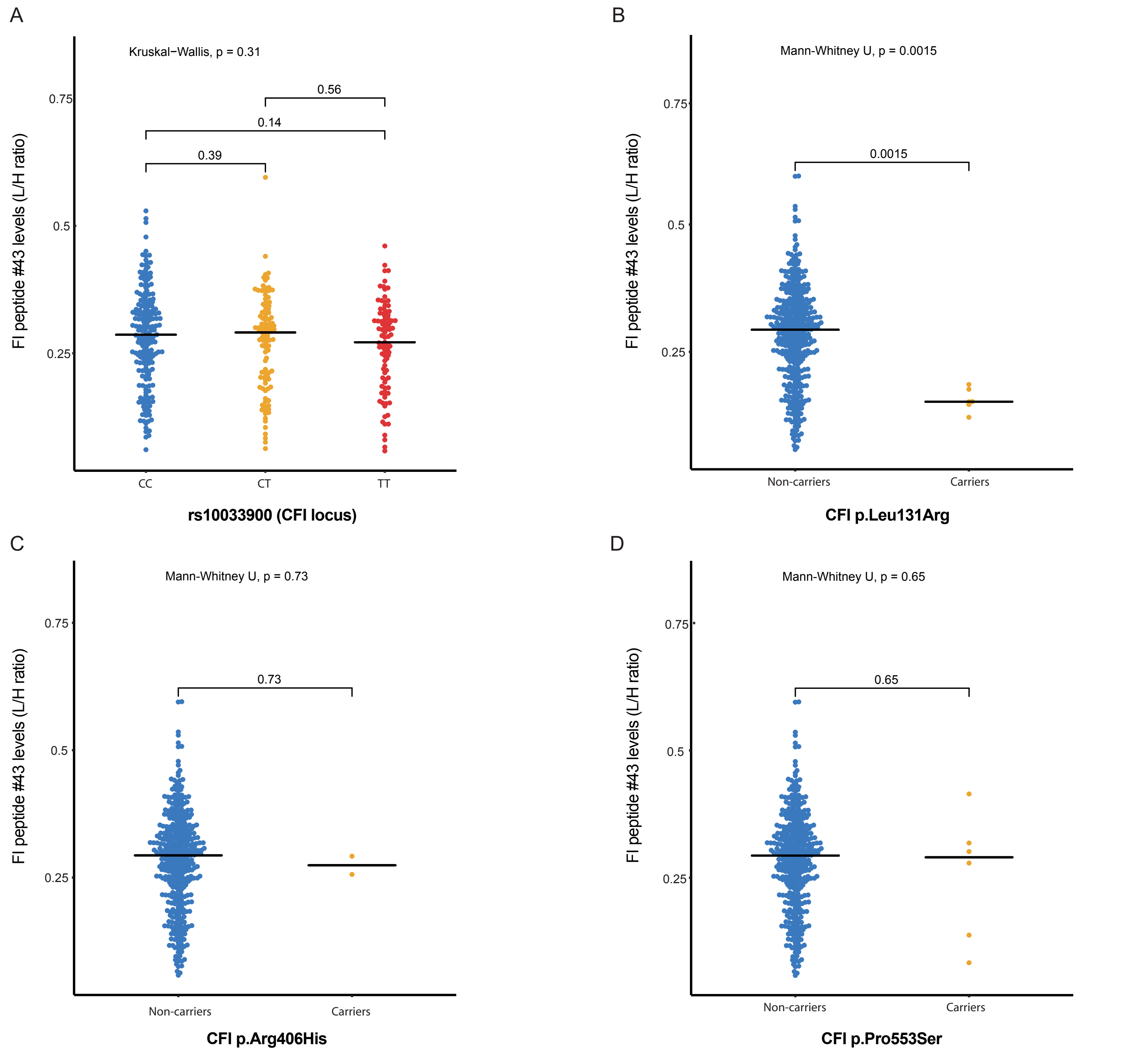

Supplement: Supplementary file 1 [file jpm-11-01256-s001.zip › SupplementaryFigureS4.pdf]
