# Supplementary figures and images for: Semi-Quantitative Multiplex Profiling of the Complement System Identifies Associations of Complement Proteins with Genetic Variants and Metabolites in Age-Related Macular Degeneration
Source: J Pers Med. 2021 Nov 25;11(12):1256. doi: 10.3390/jpm11121256 (PMC8705464; doi:10.3390/jpm11121256)

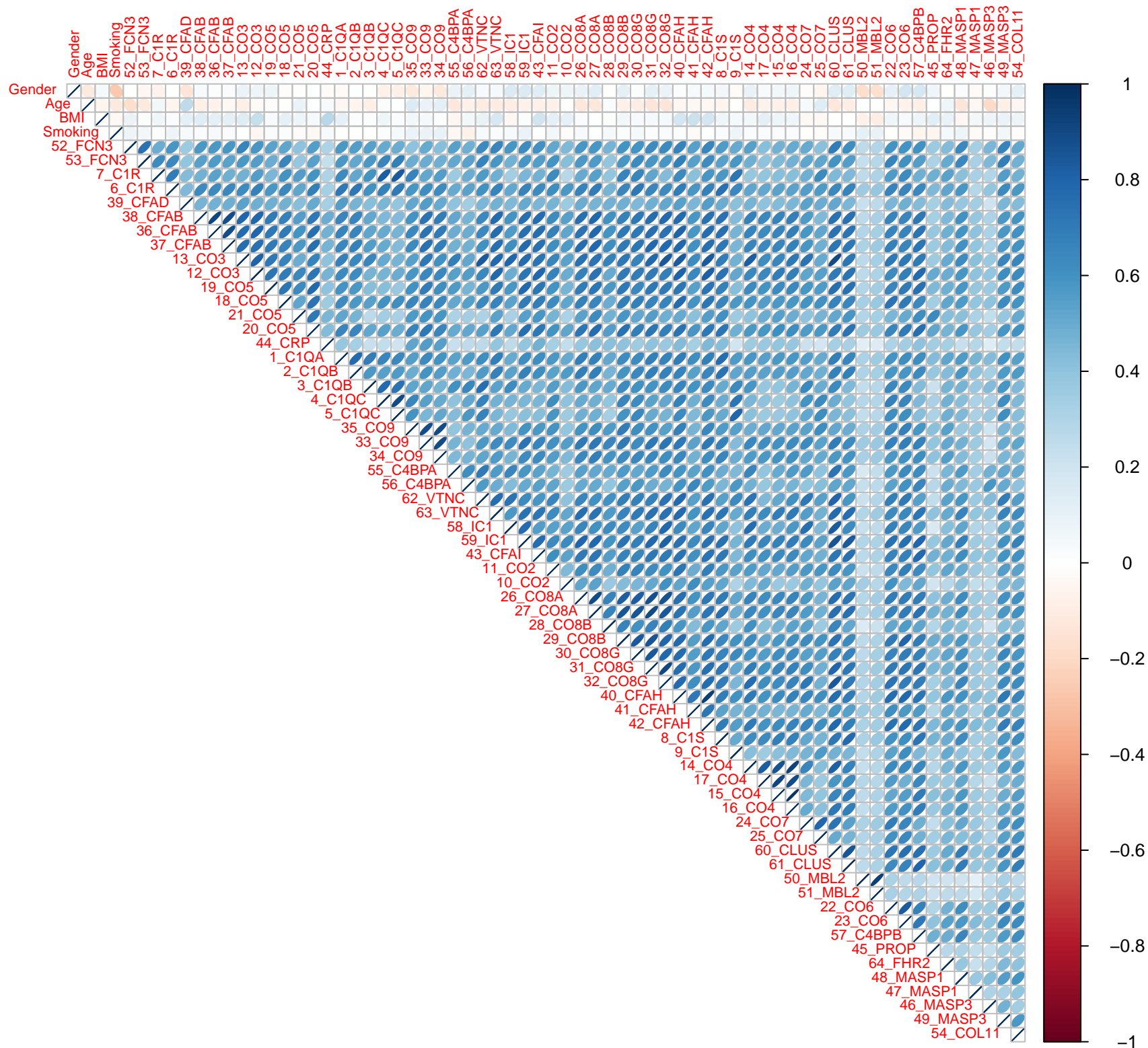

Supplement: Supplementary file 1 [file jpm-11-01256-s001.zip › SupplementaryFigureS1.pdf]
